# GC-Derived Volatile Profiling of Commercial Thyme and Oregano Labeled Products and Evaluation of Portable NIR Screening

**DOI:** 10.3390/foods15183245

**Published:** 2026-09-14

**Authors:** Necla Ozdemir-Orhan, Merve Akpinar-Uzun, Nurhan Gunes, Aziz Tekin, Ahmed Menevseoglu

**Affiliations:** 1 Department of Nutrition and Dietetics, Faculty of Health Sciences, Bitlis Eren University, Bitlis 13100, Türkiye; 2 Department of Food Engineering, Faculty of Engineering, Ankara University, Ankara 06830, Türkiye; 3 Department of Electric-Electronic Engineering, Faculty of Engineering, Sivas University of Science and Technology, Sivas 58100, Türkiye; 4 Department of Gastronomy and Culinary Arts, School of Tourism and Hotel Management, Agri Ibrahim Cecen University, Agri 04100, Türkiye

**Keywords:** GC-FID/MS, portable NIR, compositional data, chemometrics, class imbalance, herb authentication, thyme, oregano

## Abstract

Commercial aromatic herbs sold under the names thyme and oregano may encompass heterogeneous plant materials, making chemical-profile screening important even when botanical identity is unknown. This study characterized 110 commercial products marketed in Türkiye and evaluated whether portable near-infrared (NIR) spectroscopy could predict chemical-profile classes derived independently from GC-FID/MS data. Commercial labels were recorded but were not treated as verified species identities. Essential oils were measured in technical duplicate for 23 volatile compounds; replicate means were subjected to compositional data treatment, principal component analysis and Ward clustering. Two clusters were selected by the silhouette criterion (0.485), and cluster stability under technical-replicate resampling was high (median adjusted Rand index, 0.907). Cluster 1 (*n* = 17) was relatively enriched in sabinene, caryophyllene, carvacrol and delta-3-carene, whereas Cluster 2 (*n* = 93) showed higher myrcene, p-cymene, alpha-pinene and beta-pinene. Two NIR spectra were collected per sample. To prevent replicate leakage, spectra were averaged before repeated nested cross-validation; a grouped-replicate analysis was also performed. A majority-class baseline achieved 84.55% accuracy but only 0.500 balanced accuracy. The best primary model, shrinkage linear discriminant analysis, yielded a mean accuracy of 0.689, a balanced accuracy of 0.565, a macro-F1 of 0.530 and a Matthews correlation coefficient of 0.103. The grouped-replicate sensitivity analysis gave a balanced accuracy of 0.592. Thus, GC-FID/MS revealed a stable chemical-profile structure, but portable NIR did not reliably recover the minority chemical class. NIR may support exploratory triage after broader calibration, but the present data do not support stand-alone botanical authentication or definitive chemical-class assignment.

## 1. Introduction

Medicinal and aromatic plants are used as foods, flavorings and sources of bioactive compounds, but commercial names do not always map cleanly onto botanical taxa [1]. This problem is especially relevant in Türkiye, where species from Thymus, Origanum, Satureja, Thymbra and related Lamiaceae genera may be sold under the common name ‘thyme’ [2]. Essential-oil composition can also vary substantially among species, origins and processing conditions [3]. Consequently, a commercial label alone is not a valid botanical reference, and chemical profiling should be interpreted as product characterization rather than species proof.

Chromatographic profiling provides detailed information on volatile composition and can reveal chemotype-like structure within heterogeneous commercial material. Because relative abundance data are compositional, analysis in log-ratio space is preferable to direct Euclidean analysis of raw percentages [4]. Unsupervised principal component analysis (PCA) and clustering can then identify dominant axes and groups without using label prefixes as explanatory information. Such data-driven classes are useful for describing chemical diversity, but they must not be equated with botanical species in the absence of authenticated vouchers or molecular evidence.

Near-infrared spectroscopy offers rapid, non-destructive and potentially field-deployable screening. Recent work has shown encouraging results for oregano adulteration [5], herbal-tea adulteration levels [6], medicinal plant quality estimation [7], and multi-spice fraud detection [8]. Reviews, nevertheless, emphasize that preprocessing, calibration design, reference quality and external validation govern whether portable NIR models generalize [9,10]. Results obtained with deliberately prepared adulteration series or taxonomically verified materials cannot be assumed to transfer to heterogeneous, commercially labeled samples.

A further concern is validation at the correct experimental unit. When multiple spectra from one sample are split between training and test sets, models can partly recognize sample-specific signatures and overestimate performance. Hyperparameter selection and performance estimation should therefore be separated by nested cross-validation [11], and all technical replicates of a sample should remain in the same fold. With imbalanced classes, overall accuracy can also be misleading; balanced accuracy, macro-F1 and Matthews correlation coefficient (MCC) provide complementary information [12,13].

Accordingly, the objectives of this study were (i) to characterize volatile-profile heterogeneity among 110 commercial thyme- and oregano-labeled products using GC-FID/MS and compositional chemometrics, and (ii) to test, under leakage-free sample-level validation, whether portable NIR spectra could predict the GC-derived chemical-profile classes. The study does not attempt botanical differentiation because the commercial labels were not independently verified.

## 2. Materials and Methods

### 2.1. Materials

**Figure 1:**
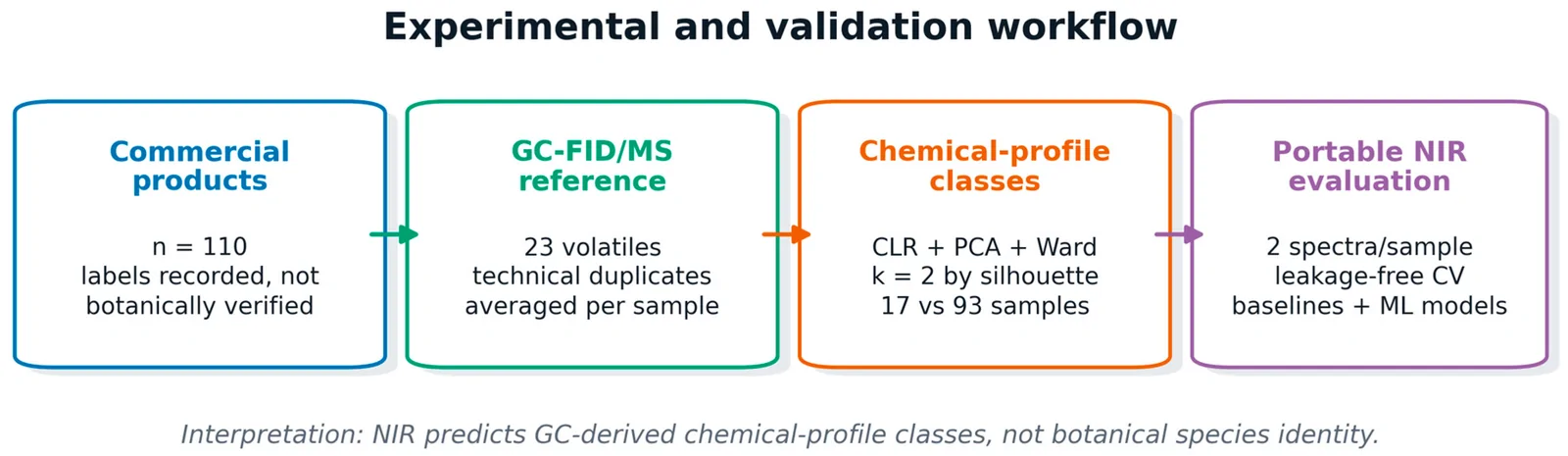
Experimental and validation workflow. Chemical-profile classes were derived solely from GC-FID/MS volatile profiles. Commercial label prefixes were not used to construct the classes.

A total of 110 products were purchased in packaged or unpackaged form from herbalists and markets in several Turkish cities, including Agri, Sivas, Samsun and Ankara. The vendors’ or packages’ commercial designation was recorded as thyme (*n* = 103) or oregano (*n* = 7). No authenticated voucher specimens, taxonomic examination or DNA-based confirmation was available. These designations were therefore treated only as market labels and sample identifiers; they were not used as botanical ground truth or as the outcome in predictive modeling. The analytical workflow is summarized in Figure 1.

### 2.2. Methods

Essential Oil Extraction: The essential oils of thyme and oregano samples were extracted using a Clevenger-type distillation apparatus, which is suitable for oils lighter than water. For this purpose, approximately 40 g of sample and 1000 mL of distilled water were added into a round-bottom flask (2000 mL), and the distillation process was initiated. The flask was first heated rapidly using a heating mantle, and after the boiling point was reached, the heating rate was reduced. The essential oil carried by water vapor in the system condensed upon contact with the condenser and was collected dropwise in the burette. The excess water collected along with the oil was returned to the flask through a discharge tube. The distillation process continued for approximately 4 h. After distillation, the essential oil sample was transferred into a tube, and anhydrous sodium sulfate (Na_2_SO_4_) was added to remove any residual water. The dehydrated essential oils were then transferred into amber vials, tightly sealed, and stored in a refrigerator at +4 °C until analysis [3].

Composition of Essential Oils: The composition of thyme and oregano essential oils was determined using a gas chromatography system coupled with a flame ionization detector (FID) (7890A, Agilent Technologies Inc., Santa Clara, CA, USA) and a mass selective detector (MSD 5975C, Agilent Technologies Inc., Santa Clara, CA, USA). A DB-624 capillary column (30 m length, 0.25 mm internal diameter, and 1.4 µm film thickness; J&W Scientific, Folsom, CA, USA) was used for the analysis, and a splitter was employed to allow simultaneous analysis of the sample by both detectors. The oven temperature was initially held at 40 °C for 5 min, then increased to 110 °C at a rate of 3 °C/min, followed by an increase to 150 °C at 4 °C/min. Subsequently, the temperature was raised to 210 °C at a rate of 10 °C/min and held for 12 min. The injector and detector temperatures were set to 250 °C. Helium was used as the carrier gas at a flow rate of 1.0 mL/min. The essential oil samples were diluted in methanol at a ratio of 1:50 and injected into the system using an appropriate split ratio. The ion source temperature was set at 230 °C, and the ionization energy was 70 eV. The identification of volatile compounds was carried out using Kováts retention indices (KI) obtained by injecting n-alkane standards into the system. Additionally, reference standards such as thymol, carvacrol, γ-terpinene, and limonene—known as the major components of thyme and oregano essential oils—were also analyzed. Furthermore, compound identification was supported by comparison with the Wiley 10/NIST 14 mass spectral libraries of the GC/MS system [14].

#### 2.2.1. Spectral Acquisitions

Near-Infrared (NIR) Spectroscopy: Near-infrared (NIR) spectra of the samples were collected using a micro-NeoSpectra spectrometer (Si-Ware Systems, Cairo, Egypt). The spectrometer operates over the spectral range of 7400–3920 cm^−1^ and is equipped with an InGaAs photodetector. Spectra were acquired in absorbance mode at a spectral resolution of 16 cm^−1^. A scanning time of 15 s was used for each measurement to improve the signal-to-noise ratio. Each sample was measured twice, and the replicate spectra were used for subsequent analysis.

#### 2.2.2. GC Compositional Analysis and Data-Driven Clustering

Technical duplicates were averaged at the sample level before the main analysis. For each volatile compound, zero values were replaced by one-half of that compound’s smallest positive sample mean. Each profile was closed to sum to one and transformed by the centered log-ratio (CLR) transformation. PCA was applied to the CLR matrix. Ward hierarchical clustering with Euclidean distance was evaluated for k = 2 to 10 clusters; the solution with the largest average silhouette coefficient was selected [15]. Sample codes and the K, BK and OR prefixes were not included in PCA or clustering.

Cluster robustness to technical-replicate choice was assessed in 500 iterations. In each iteration, one of the two GC measurements was randomly selected for each sample; the zero replacement and CLR transformation were repeated, and Ward clustering was rerun. Agreement with the reference solution was quantified by the adjusted Rand index (ARI; [16]). Cluster-wise compound summaries used medians, Mann–Whitney tests with Benjamini–Hochberg adjustment and Cliff’s delta. Because the clusters were defined from the same volatile variables, these comparisons were interpreted as post hoc descriptive characterization rather than independent hypothesis confirmation.

#### 2.2.3. Leakage-Free NIR Modeling and Performance Assessment

The GC-derived Ward cluster was the only prediction target. The primary analysis averaged the two NIR spectra of each product before any train-test splitting, so the effective sample size was 110. Exploratory spectral PCA was performed after standard normal variate (SNV) transformation. Replicate agreement was summarized by the within-sample Pearson correlation and root-mean-square absorbance difference.

Four classifiers were compared: a majority-class baseline, shrinkage linear discriminant analysis (LDA) with equal class priors, partial least-squares discriminant analysis (PLS-DA), and a class-weighted radial-basis-function support vector machine (RBF-SVM). Candidate preprocessing comprised raw absorbance, SNV, a Savitzky–Golay first derivative (11-point window, second-order polynomial), and SNV followed by the same derivative. Model hyperparameters and preprocessing were selected only within the training data by four-fold stratified inner cross-validation optimizing balanced accuracy.

Generalization performance was estimated with repeated stratified five-fold outer cross-validation (10 repeats; 50 outer test folds). A single stratified five-fold out-of-fold prediction set was additionally retained for an interpretable confusion matrix. Performance measures were accuracy, balanced accuracy, macro-F1, MCC, Cluster 1 sensitivity and Cluster 2 specificity. As a sensitivity analysis, models were also evaluated on all 220 spectra with five repeats of stratified group five-fold cross-validation; both spectra from a product were always assigned to the same fold, and replicate predictions were averaged to a sample-level decision. For the grouped-replicate sensitivity analysis, a continuous decision score was generated for each held-out spectrum. The two replicate decision scores for each product were averaged, and the product was assigned to Cluster 1 when the mean score was greater than or equal to zero and to Cluster 2 otherwise; predicted probabilities and hard class labels were not averaged.

Analyses were performed in Python 3.12 using NumPy 2.3.5, pandas 2.2.3, SciPy 1.17.0 and scikit-learn 1.8.0. Random seeds and fold assignments were fixed for reproducibility.

## 3. Results

### 3.1. Volatile-Profile Heterogeneity

The 110 sample-level volatile profiles were chemically heterogeneous. The greatest between-sample standard deviations were observed for carvacrol (6.34 percentage points), myrcene (5.44), gamma-terpinene (4.08), p-cymene (3.83), sabinene (2.02) and thymol (1.38). Carvacrol reached 62.42% in K85; the highest sample means for sabinene, myrcene, gamma-terpinene and thymol occurred in K97 (8.80%), K55 (30.60%), K6 (30.41%) and K82 (10.94%), respectively. These summaries describe market-product variation; technical duplicates were not treated as independent biological replicates.

### 3.2. PCA, Cluster Selection and Cluster Stability

**Figure 2:**
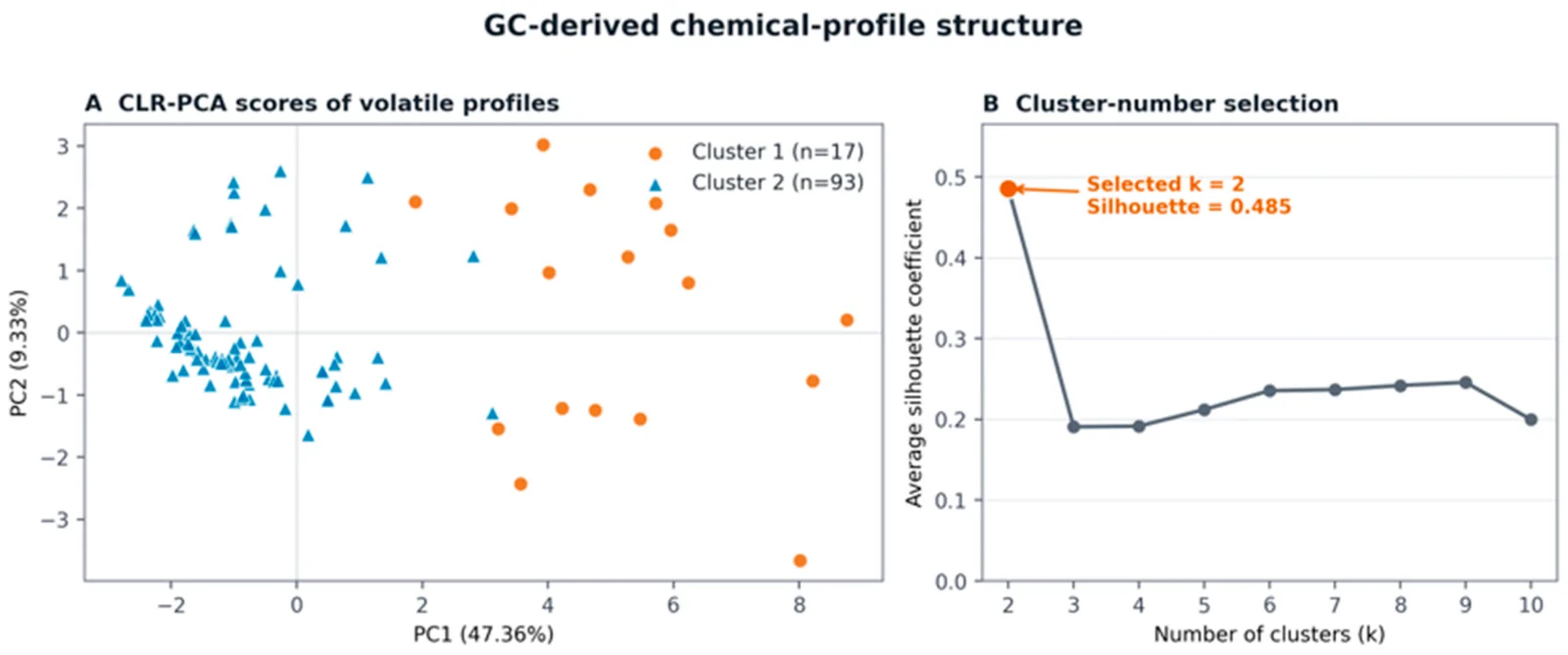
GC-derived chemical-profile structure. (**A**) PCA scores from CLR-transformed sample-mean volatile profiles. (**B**) Average silhouette coefficients for Ward solutions containing 2–10 clusters.

For the CLR-transformed volatile profiles, PC1, PC2 and PC3 explained 47.36%, 9.33% and 7.39% of total variance, respectively (64.08% cumulative). Ward clustering produced its largest silhouette coefficient at k = 2 (0.485; Figure 2). Cluster 1 contained 17 samples, and Cluster 2 contained 93. The reference solution was robust to which GC technical replicate was selected (median ARI across 500 iterations = 0.907). This stability concerns the analytical cluster structure; it does not establish taxonomic identity.

All 17 Cluster 1 products carried a thyme-related commercial label. Cluster 2, nevertheless, contained 86 thyme-labeled products as well as all seven oregano-labeled products. Thus, commercial naming did not provide a one-to-one mapping to the GC classes, and Cluster 2 cannot be interpreted as an oregano species class.

### 3.3. Volatile Compounds Discriminating the Two Clusters

Cluster 1 was most strongly characterized by higher levels of sabinene and caryophyllene, together with higher levels of delta-3-carene and a modestly higher carvacrol median. Cluster 2 showed higher levels of myrcene, p-cymene, alpha-pinene and beta-pinene. The effect-size pattern is summarized in Table 1. The post hoc q-values are descriptive because the same volatile matrix generated the clusters.

### 3.4. NIR Repeatability and Unsupervised Spectral Structure

**Figure 3:**
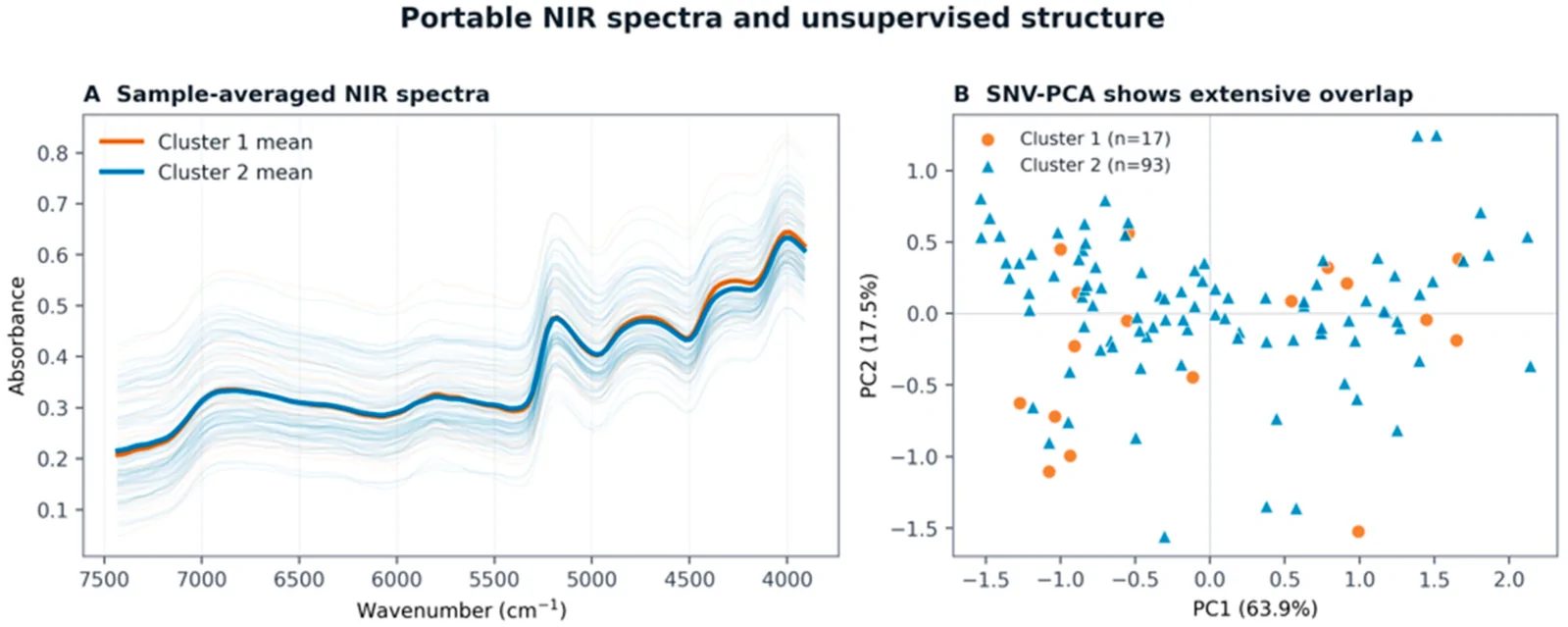
Portable NIR spectra and unsupervised structure. (**A**) Sample-averaged spectra; thin lines show individual sample means, and thick lines show class means. (**B**) PCA scores after SNV preprocessing.

Technical NIR replicate agreement was high (mean within-sample Pearson r = 0.9967; median = 0.9984; minimum = 0.9704), and the mean replicate root-mean-square absorbance difference was 0.0351. Therefore, poor class prediction was not attributable simply to unstable repeat measurements. Broad spectral features occurred near 4300–4400, 5100–5200, 5700–5900 and 6900 cm^−1^, regions commonly associated with C-H and O-H combination or overtone bands in plant matrices [10]. After SNV preprocessing, the first two PCA components explained 63.9% and 17.5% of the variance. The two GC-derived groups overlapped extensively in the PCA score space (Figure 3).

### 3.5. Leakage-Free Prediction of GC-Derived Classes

**Figure 4:**
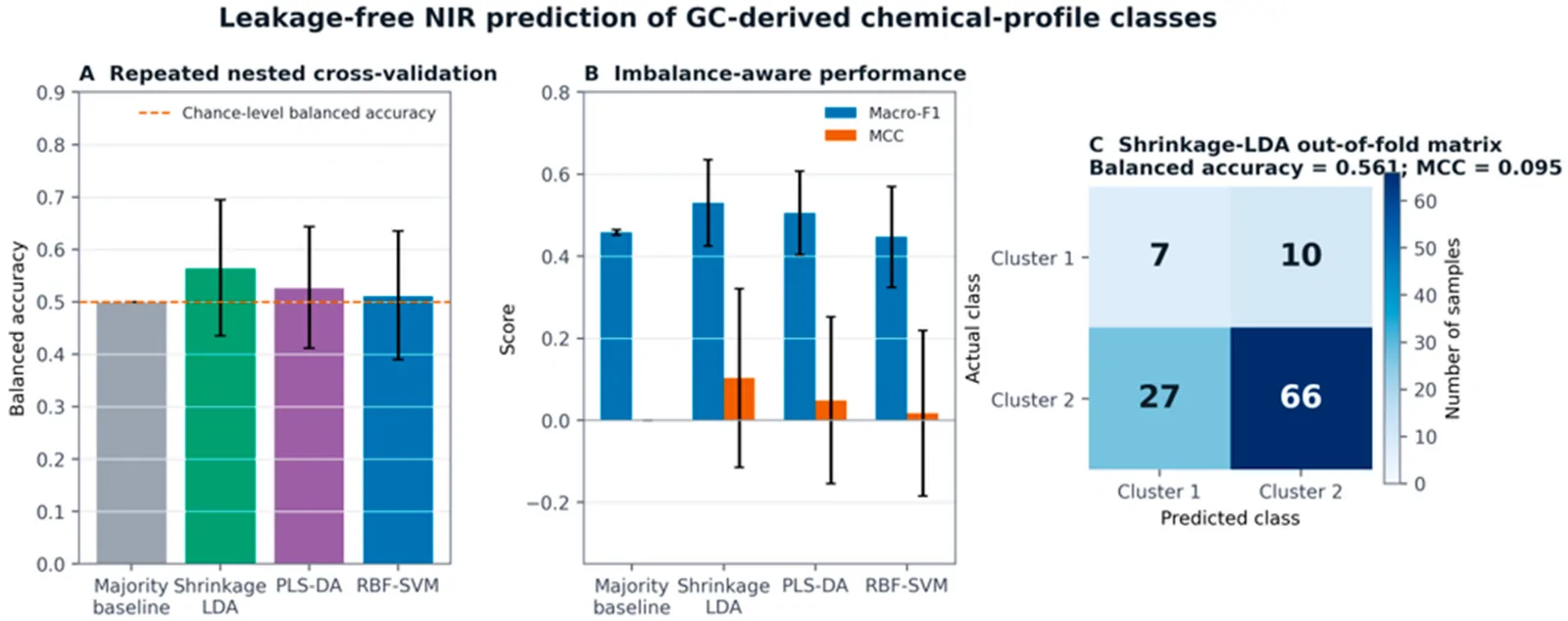
Leakage-free NIR prediction of GC-derived chemical-profile classes. (**A**,**B**) summarize mean performance across 50 outer test folds, with error bars representing the standard deviation. (**C**) shows pooled out-of-fold predictions from a separate single stratified five-fold run in which each product was predicted once (balanced accuracy = 0.561; MCC = 0.095); these single-run values are distinct from the 50 outer-fold averages reported in Table 2.

Because 93 of the 110 samples belonged to Cluster 2, a classifier that always predicted Cluster 2 achieved an accuracy of 0.845. Its balanced accuracy was 0.500, macro-F1 was 0.458 and MCC was 0, demonstrating why overall accuracy alone was not an appropriate primary measure. Across 50 outer folds of the repeated nested sample-level analysis, shrinkage LDA had the highest mean balanced accuracy (0.565), macro-F1 (0.530) and MCC (0.103), with a mean accuracy of 0.689. PLS-DA and RBF-SVM did not improve balanced performance (Table 2; Figure 4). In the separate single five-fold out-of-fold prediction set, shrinkage LDA correctly identified 7 of 17 Cluster 1 products and 66 of 93 Cluster 2 products; balanced accuracy was 0.561, and MCC was 0.095. These single-run values are reported only to accompany the confusion matrix and are distinct from the repeated 50-outer-fold summary estimates in Table 2.

The grouped-replicate sensitivity analysis, which retained both spectra from a sample in the same fold, gave a slightly higher but still limited mean balanced accuracy of 0.592 for shrinkage LDA (macro-F1 = 0.567; MCC = 0.165). Fold-to-fold variability was substantial, and the 2.5th–97.5th percentile interval of balanced accuracy was 0.389–0.792. These results indicate weak and unstable recovery of the minority chemical-profile class.

## 4. Discussion

The GC-FID/MS data revealed two stable, data-driven chemical-profile groups within the commercial collection. The largest contrast was a sabinene–caryophyllene-rich profile versus a myrcene–p-cymene–pinene-rich profile, with carvacrol remaining abundant in both groups. This pattern is compatible with the broad chemical diversity expected within commercial Lamiaceae materials, and it supports the use of multivariate chemical fingerprints rather than a single-marker rule. Other recent studies have likewise used combined fingerprints and discriminant analysis to distinguish Lamiaceae materials [17,18]. The overlap in GC PCA and the unbalanced 17/93 partition nevertheless suggest a continuum with one smaller extreme group rather than two taxonomically discrete populations.

Crucially, the GC groups were chemical classes, not thyme and oregano species classes. All oregano-labeled products fell in Cluster 2, but so did most thyme-labeled products. Without authenticated plant material, the commercial designations cannot validate botanical identity. The redefined objective, therefore, aligns the inference with the actual evidence: the study characterizes commercial chemical diversity and tests whether NIR can reproduce that chemical grouping. This distinction is essential for avoiding an unsupported authentication claim.

Portable NIR showed excellent measurement repeatability but weak predictive discrimination. This combination is informative: the limiting factor was not gross instrument instability, but the modest relationship between the NIR matrix and the GC-derived minority profile under the present sampling design. NIR responds to broad overtone and combination bands from the whole powdered matrix, whereas the target was defined from relative concentrations of 23 volatile oil constituents. Matrix composition, moisture, particle size and non-volatile constituents may therefore dominate spectral variance without mapping uniquely onto volatile chemotypes.

Encouraging NIR results reported for oregano, paprika, cumin, herbal teas and designed spice-adulteration series [5,6,8,19,20] were obtained with different targets and, often, more controlled class definitions. In contrast, the present reference classes were unbalanced and reflected a continuous commercial chemical space. The comparison shows why validation design and target definition matter as much as algorithm choice. The nonlinear RBF-SVM did not outperform shrinkage LDA, indicating that model complexity could not compensate for limited minority samples and spectral overlap.

For practical use, the current NIR model should not be deployed as a stand-alone release test. A future calibration study should collect botanically authenticated voucher material across species, origins, seasons and processors; balance the chemical classes; record independent production lots; and reserve an external prospective test set. Orthogonal botanical confirmation, such as expert taxonomy and DNA barcoding, would permit a separate and legitimate species-authentication objective. Until then, portable NIR may be investigated as a low-cost triage tool that flags unusual material for confirmatory GC or molecular analysis, preferably with an explicit reject/uncertain category.

## 5. Conclusions

GC-FID/MS combined with compositional PCA and Ward clustering identified a reproducible two-group structure among 110 commercial products labeled as thyme or oregano. The groups represented contrasting volatile profiles, not verified botanical species. When NIR performance was re-evaluated at the sample level without cross-fold technical-replicate leakage, using a majority baseline and imbalance-aware metrics, shrinkage LDA achieved only modest balanced accuracy and MCC, while PLS-DA and RBF-SVM did not improve the results. The strong agreement between duplicate NIR spectra indicates that the main limitation was weak class information rather than measurement repeatability. Portable NIR may therefore have value for exploratory triage after the expansion of the calibration set, but the present model is not adequate for stand-alone botanical authentication or definitive chemical-profile classification.

## Figures and Tables

**Table 1:** Post hoc characterization of the two GC-derived clusters.

Volatile Compound	Cluster 1 Median (%)	Cluster 2 Median (%)	Cliff’s Delta	BH-Adjusted q	Interpretation
Sabinene	4.466	0.000	0.968	5.23 × 10^−12^	Higher in Cluster 1
Caryophyllene	1.345	0.000	0.934	6.10 × 10^−15^	Higher in Cluster 1
Myrcene	5.268	12.913	−0.922	1.34 × 10^−8^	Higher in Cluster 2
alpha-Pinene	0.194	1.250	−0.891	2.66 × 10^−8^	Higher in Cluster 2
beta-Pinene	0.109	0.768	−0.891	2.66 × 10^−8^	Higher in Cluster 2
p-Cymene	3.740	9.869	−0.882	3.15 × 10^−8^	Higher in Cluster 2
Carvacrol	57.229	54.657	0.629	9.11 × 10^−5^	Higher in Cluster 1
delta-3-Carene	0.588	0.000	0.541	8.45 × 10^−6^	Higher in Cluster 1
Thymol	5.021	4.305	0.351	0.0316	Higher in Cluster 1
gamma-Terpinene	9.656	10.547	−0.195	0.246	Not significant

**Table 2:** Leakage-free NIR performance for prediction of GC-derived chemical-profile classes.

Model	Accuracy	Balanced Accuracy	Macro-F1	MCC	Cluster 1 Sensitivity
Majority baseline	0.845 (0.022)	0.500 (0.000)	0.458 (0.007)	0.000 (0.000)	0.000
Shrinkage LDA	0.689 (0.090)	0.565 (0.130)	0.530 (0.105)	0.103 (0.218)	0.382
PLS-DA	0.709 (0.107)	0.528 (0.116)	0.506 (0.102)	0.049 (0.203)	0.262
RBF-SVM	0.644 (0.185)	0.512 (0.123)	0.447 (0.123)	0.017 (0.202)	0.330
Grouped-replicate LDA	0.715 (0.091)	0.592 (0.113)	0.567 (0.103)	0.165 (0.203)	0.417

For the majority baseline, shrinkage LDA, PLS-DA, and RBF-SVM, values are presented as mean (SD) across 50 outer test folds (10 repeats by five folds), except that Cluster 1 sensitivity is shown as the mean only. The grouped-replicate LDA row summarizes 25 outer test folds (five repeats by five stratified group folds). All non-grouped rows use spectra averaged within the product before splitting.

## Data Availability

The raw GC-FID/MS and NIR data, sample-level GC-derived cluster labels, analysis code, preprocessing and model specifications, random seeds, and fixed inner- and outer-cross-validation fold assignments are available from the corresponding author upon reasonable request.

## References

[1] Christaki E., Bonos E., Giannenas I., Florou-Paneri P. (2012). Aromatic plants as a source of bioactive compounds. Agriculture.

[2] Bozdemir C. (2019). Türkiye’de yetisen kekik türleri, ekonomik önemi ve kullanim alanlari. Yuz. Yıl Univ. J. Agric. Sci..

[3] Ozdemir N., Ozgen Y., Kiralan M., Bayrak A., Arslan N., Ramadan M.F. (2018). Effect of different drying methods on the essential oil yield, composition and antioxidant activity of *Origanum vulgare* L. and *Origanum onites* L. J. Food Meas. Charact..

[4] Aitchison J. (1982). The statistical analysis of compositional data. J. R. Stat. Soc. Ser. B.

[5] Sammarco G., Alinovi M., Fiorani L., Rinaldi M., Suman M., Lai A., Puiu A., Giardina I., Pollastrone F. (2023). Oregano herb adulteration detection through rapid spectroscopic approaches: Fourier transform-near infrared and laser photoacoustic spectroscopy facilities. J. Food Compos. Anal..

[6] Steidle Neto A.J., de Carvalho Lopes D. (2024). Chemometrics coupled with near infrared spectroscopy for detecting adulteration levels in herbal teas. J. Food Compos. Anal..

[7] Sing D., Banerjee S., Mallik R., Yonzone U.A., Hazarika A.K., Majumdar K., Bandyoypadhyay R. (2024). Rapid and non-destructive quality estimation of cinchona, Andrographis paniculata, and black pepper using a portable NIR spectroscopy measuring device. Microchem. J..

[8] Schumer N.G., Ahmed M.W., Rausch K., Singh V., Kamruzzaman M. (2025). Chemometric-based approach for economically motivated fraud detection in organic spices via NIR spectroscopy. J. Food Compos. Anal..

[9] Chen R., Liu F., Zhang C., Wang W., Yang R., Zhao Y., Peng J., Kong W., Huang J. (2023). Trends in digital detection for the quality and safety of herbs using infrared and Raman spectroscopy. Front. Plant Sci..

[10] Shawky E., Nahar L., Nassief S.M., Sarker S.D., Ibrahim R.S. (2024). Spice authentication by near-infrared spectroscopy: Current advances, limitations, and future perspectives. Trends Food Sci. Technol..

[11] Varma S., Simon R. (2006). Bias in error estimation when using cross-validation for model selection. BMC Bioinform..

[12] Brodersen K.H., Ong C.S., Stephan K.E., Buhmann J.M. (2010). The balanced accuracy and its posterior distribution. Proceedings of the 20th International Conference on Pattern Recognition 2010, Istanbul, Turkey, 23–26 August 2010.

[13] Chicco D., Jurman G. (2020). The advantages of the Matthews correlation coefficient over F1 score and accuracy in binary classification evaluation. BMC Genom..

[14] Kiralan M. (2014). Changes in Volatile Compounds of Black Cumin (*Nigella sativa* L.) Seed Oil During Thermal Oxidation. Int. J. Food Prop..

[15] Rousseeuw P.J. (1987). Silhouettes: A graphical aid to the interpretation and validation of cluster analysis. J. Comput. Appl. Math..

[16] Hubert L., Arabie P. (1985). Comparing partitions. J. Classif..

[17] Tarighat M.A., Abdi G., Tarighat F.A., Bayatiyani K.S. (2023). Authentication and identification of Lamiaceae family with cyclic voltammetry fingerprint-PCA-LDA and determination of the used phenolic contents for classification using chromatographic analyses. Talanta.

[18] Kucharska-Ambrozej K., Karpinska J., Ratkiewicz A. (2026). Performance of classification tools for small Lamiaceae herb samples. Results Chem..

[19] Oliveira M.M., Cruz-Tirado J.P., Roque J.V., Teofilo R.F., Barbin D.F. (2020). Portable near-infrared spectroscopy for rapid authentication of adulterated paprika powder. J. Food Compos. Anal..

[20] Cruz-Tirado J.P., de Franca R.L., Tumbajulca M., Barraza-Jauregui G., Barbin D.F., Siche R. (2023). Detection of cumin powder adulteration with allergenic nutshells using FT-IR and portable NIRS coupled with chemometrics. J. Food Compos. Anal..

